# *Prakriti* elucidates the inter-individual variability in coronary artery disease risk-predicting biomarkers: A tertiary care hospital-based case control study

**DOI:** 10.1016/j.jaim.2025.101178

**Published:** 2025-08-19

**Authors:** Pamila Dua, Bhavana Prasher, Sandeep Seth, Shivam Pandey, Subir Kumar Maulik, K.H. Reeta

**Affiliations:** a Department of Pharmacology, All India Institute of Medical Sciences, Ansari Nagar, New Delhi, 110029, India; b Department of Ayurgenomics, Institute of Genomics and Integrative Biology, CSIR, South Campus, Delhi, 110025, India; c Department of Cardiology, All India Institute of Medical Sciences, Ansari Nagar, New Delhi, 110029, India; d Department of Biostatistics, All India Institute of Medical Sciences, Ansari Nagar, New Delhi, 110029, India; e Department of Pharmacology, All India Institute of Medical Sciences, Ansari Nagar, New Delhi, 110029, India

**Keywords:** Coronary artery disease, Biomarkers, Phenotype, Ayurveda, *Prakriti*

## Abstract

**Background:**

Several biochemical tests and biomarkers are well-known for the assessment of risk towards coronary artery disease (CAD). However, conflicting results pose a significant challenge probably due to phenotypic heterogeneity. In Ayurveda, individuals are classified into phenotypes- *Prakriti,* which helps in predicting an individual's susceptibility to disease, its prognosis and selection of therapy. In the present study, an attempt was made to overcome this challenge with an aim to identify the association between different constitution types as mentioned in Ayurveda with biochemical markers for precisely predicting the risks for CAD.

**Methods:**

200 clinically stable CAD patients and 100 healthy controls were recruited from the Cardiology OPD, AIIMS, New Delhi, India. A comprehensive set of tests to incorporate various aspects of CAD pathophysiology was performed. Assessment of *Prakriti* was done clinically and with AI/ML algorithm based validated questionnaire.

**Results:**

The monocyte-lymphocyte ratio (MLR), fasting blood sugar, urea, creatinine, uric acid, and NT-pro BNP were significantly higher in CAD patients as compared to healthy controls. *Prakriti* stratification revealed maximum number of patients with *Kapha Prakriti*. MLR and IL-6 (associated with inflammatory and peripheral endothelial dysfunction) were high in *Vata* patients; diabetic control (associated with plaque instability and malfunctioned RAAS) was poor in *Kapha* patients and NT-pro BNP (associated with myocardial hypoxia) was higher in *Pitta* patients.

**Conclusion:**

Though, several biochemical parameters were associated with risks for CAD, *Prakriti* classification provided more insights into the precise risks. This dual approach may help in guiding personalized treatment options in CAD management.

## Highlights

• Several biochemical tests and biomarkers exist for risk assessment of CAD.
• Conflicting results pose a significant challenge due to phenotypic heterogeneity.
• In Ayurveda, *Prakriti* may help predict an individual's susceptibility to disease.
• 200 CAD patients & 100 healthy controls underwent dual approach of risk assessment.
• *Prakriti* classification provided more insights into the precise risks of CAD.

## 1 Introduction

**Fig. 1:**
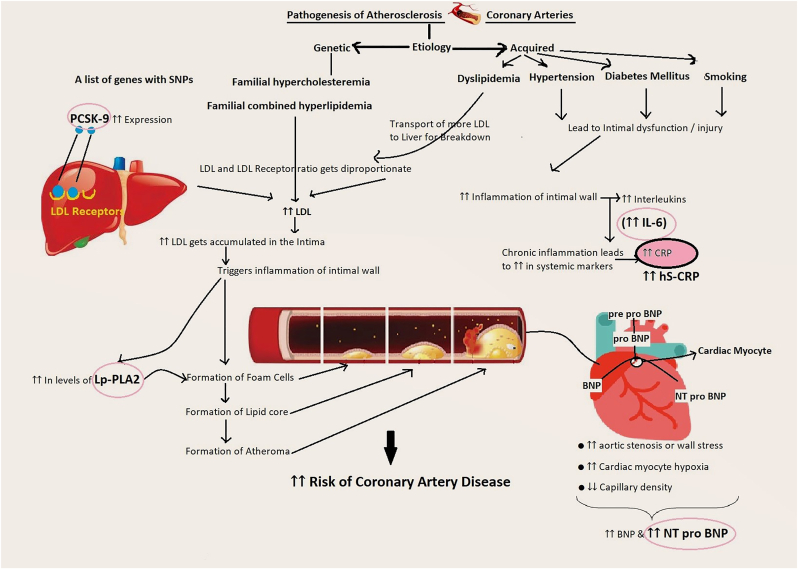
Diagrammatic presentation of pathogenesis and markers involved in CAD.

Coronary artery disease (CAD) has emerged as one of the leading causes of death and disability worldwide [1]. CAD, also known as ischemic heart disease, is a condition that occurs when the coronary arteries supplying oxygenated blood to the heart muscles become narrowed or blocked, leading to reduced blood flow to the heart [2]. Several biochemical tests and biomarkers are established to assess the severity [3]. One of the major components in the origin and severity of the disease could be oxidative stress. Increased oxidant production and impaired endogenous antioxidant systems result in oxidative stress [4]. Several pathological events cause the production of reactive oxygen species by activating mitochondrial and NADPH oxidases [5]. Activation of redox-sensitive signaling pathways promotes the expression of cytokines and growth factors [6] resulting in the first step of atherogenesis. The endothelium becomes structurally and functionally impaired [7]. Further, the pathology involved at various steps in development of CAD may involve platelet activation [8], inflammation [9], plaque rupture or instability [10], ischemia [11] and myocardial dysfunction [12]. Stress may be presented with multiple biomarkers like metabolic [13], hemodynamic, and inflammatory [14]. Various biomarkers are associated with the different stages of CAD [15]. Diabetes, tobacco, smoking, hypertension, lipid disorders and genetic factors (family history of ischemic heart disease), age and obesity predispose to the development of atherosclerosis and CAD [16]. Atherosclerosis is a sequence of endothelial dysfunction [17] originating with formation of foam cells with fatty streaks and plaque formation followed by calcification and blockage of coronary arteries [18]. Inflammatory cells release cytokines, which contribute to the destabilization of plaques, creating a rupture-prone environment. After a plaque rupture or myocardial infarction, the body initiates healing processes involving inflammation, tissue repair, and scar formation [19]. The affected vessel may undergo remodeling, which can affect its structure and function. The complete cascade of pathogenesis of CAD is presented in Fig. 1.

In the present study, to capture a panoramic view of whole cascade of this network, we have selected established biochemical parameters and biomarkers for CAD which are representative parameters of the various steps in the disease pathophysiology.

Ayurveda is an ancient system of medicine practiced in India [20]. Ayurveda phenotypically classifies individuals into seven broad constitution types termed as *Prakriti* [21]. These can be referred as personality, basic nature, or the state of health of an individual. As per Ayurveda, everyone is born with a specific *Prakriti*. Within our bodies, there exists a constant equilibrium of *Tridoshas* known as *Vata (V), Pitta (P),* and *Kapha (K).* These three elemental forces or physiological components are inherent in all cells of our body. They play a crucial role during conception, help in shaping the overall bodily characteristics and establishing an individual's constitution. The primary constitution types such as V, P, K, VP, PK, VK, or VKP, are created depending on the respective proportions of these *doshas* [22].

Further, these readily recognizable phenotypes, respond differently to diet, drugs, and external environment as well as vary in predisposition to specific diseases, therapy, and life-style regime. The concept is thus claimed to be useful in predicting an individual's susceptibility to a particular disease, prognosis of illness and selection of therapy. The assessment of *Prakriti* of an individual involves a comprehensive analysis encompassing anatomical, physiological, and psychological traits. There are different methods for assessing *Prakriti* including clinical examinations and medical learning algorithm based validated questionnaire. Studies have also explained about the susceptibility of different disorders in specific type of *Prakriti* [23]. *Prakriti* based assessment may thus be useful in precision medicine for the stratification of endo-phenotypes in healthy and diseased population.

The present study aimed to identify the biochemical tests and biomarkers which can be used for the assessment of severity in CAD in general and in the present set of population. Further, we proposed to understand if individuals with different *Prakritis* show differences in CAD predisposition and if a predominant type of *Prakriti* individuals predisposed to CAD were different from healthy individuals or other diseased population.

## 2 Methods

### 2.1 Study design

The present study was an observational case control study, which was conducted in a single tertiary care center at All India Institute of Medical Sciences (AIIMS), New Delhi, India. Written approval from the Institute ethics committee (IEC510/05/10, 2018, RP-40/2018) was obtained before commencement of the study. The study was carried out according to the ICMR's ethical guidelines for biomedical research on human subjects (2017) and was prospectively registered in Clinical Trial Registry of India (CTRI/2019/01/016866). Written informed consent was obtained from participants before enrolment.

### 2.2 Sample size calculation

The present study aimed to analyze the association of different biochemical parameters and biomarkers in different predominant *Prakriti* types. A previously published study revealed a strong relation of risk factors like diabetes and hypertension in different *Prakriti* types and demonstrated predisposition to cardiovascular disorders in specific *Prakriti* individuals [24]. In the present study, we confined our focus only on CAD patients. We hypothesized that the proportion of healthy controls would be 40 %. Therefore, with an allocation ratio of 2:1 (CAD patients: controls), to achieve 80 % power, at 5 % level of significance, the sample size with continuity correction was calculated as 160 CAD patients and 80 controls. Taking into consideration an attrition rate of approximately 25 %, the sample size for the study was fixed at 200 CAD patients and 100 healthy controls.

### 2.3 Study population and eligibility

The first participant was recruited on May 19, 2019. The recruitment of all participants of both groups was completed by May 1, 2023. Participants in group I (CAD patients) were screened from Cardiology OPD, AIIMS, New Delhi, India, and healthy controls (group II) were screened from the attendants of the patients and other healthy volunteers working in the institute. Individuals diagnosed with CAD on standard drug regimen, clinically stable since last 3 months, both genders, with age between 18 and 75 years and willing to participate were included. Participants enrolled in any other study, diagnosed with renal failure or liver dysfunction, and who had undergone any recent interventions were excluded.

**Fig. 2:**
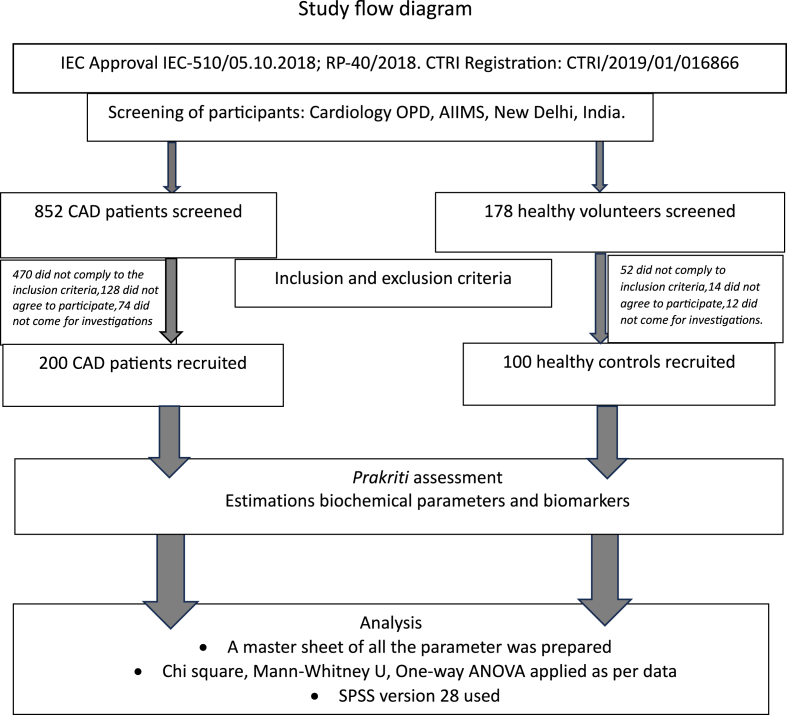
Study flow diagram.

Overall, 852 patients were screened for recruitment of 200 stable CAD patients in group I and 178 healthy volunteers were screened for recruitment of 100 healthy controls in group II. The study flow diagram is depicted in Fig. 2.

### 2.4 Stability assessment

The clinical stability criteria of all enrolled CAD patients were based on their clinical assessment, Seattle angina score [25] and ejection fraction [26,27] in echocardiography. Permission was obtained to use the Seattle angina questionnaire [28]. Stable CAD is a noncommunicable global burden, and is 125.129 as per the International Classification of Diseases, Tenth Revision [29].

### 2.5 Prakriti assessment

The phenotypic data analysis to identify *Prakriti* was performed through detailed history and examination done by the physician followed by a software-based analysis. CSIR-IGIB questionnaire was used for the *Prakriti* assessment. This is a validated questionnaire and had been used in earlier built models. It has a list of questions dealing with different domains like demographic, anatomical, physiological, behavioral, and emotional attitude of the person [23]. The data gathered manually were transferred to the software which works on AI/ML based algorithm. Based on the score of all features, the software analyzes the data and interprets the score as per the dominant proportions for identification of the predominant *Prakriti*.

### 2.6 Blood sampling and processing

Around 8 ml of peripheral blood sample of all recruited participant was collected in labelled vacutainers as per ICMR standard guidelines [30]. 4 ml of blood sample was used for all biochemical estimations and 4 ml of the sample was processed into serum/plasma for their further use in estimations of biomarkers. All the aliquots of processed samples were stored in −80^0^C refrigerator until analysis within three months.

### 2.7 Estimation of biochemical parameters and biomarkers

The selected parameters were established biochemical parameters and biomarkers for CAD. These included hemogram, differential cell count, monocyte to lymphocyte ratio (MLR) [31], lipid profile [32], fasting blood sugar (FBS) [33], glycosylated hemoglobin (HbA1c) [34], liver function test (LFT) [35], kidney function test (KFT) [36], C-reactive protein (CRP) [37], interleukin-6 (IL- 6) [38], proprotein convertase subtilisin/kexin type-9 (PCSK9) [39], lipoprotein-associated phospholipase A2 (LpPLA2) [40], malondialdehyde (MDA) [41], catalase, glutathione (GSH) [42], superoxide dismutase (SOD) [43], and N-terminal pro B-type natriuretic peptide (NT-pro BNP) [44]. All the estimations were performed as per protocol. Further, biochemical estimations for complete hemogram, LFT, KFT lipid profile, PT-INR, blood sugar fasting, HbA1C, etc. were carried out using automated analyzer and standardized kits in Centralized Laboratory Facility, Cardiothoracic and Neuroscience Center, AIIMS, New Delhi.

Estimations of biomarkers like IL-6 (Cayman CHEMICAL catalog no. 501030), SOD (Cayman CHEMICAL catalog no.706002), MDA (Cayman CHEMICAL catalog no. 700870), catalase (Cayman CHEMICAL catalog no. 707002), GSH (Cayman CHEMICAL catalog no.703002) were done using ELISA as per the manufacturer's protocol. Biomarkers like PCSK9, LpPLA2, CRP were estimated through Multiplex ELISA (R&D SYSTEMS Luminex Discovery Assay Catalog no. LXSAHM-03) with Luminex 100/200 Machine and NT-pro BNP using QUIDEL Triage NT-pro BNP TEST.

### 2.8 Statistical analysis

Chi square, Mann-Whitney U, and post hoc analysis tests were applied as per the types of data. Statistical Package for Social Sciences (SPSS) version 28.0 was used for analysis. Univariate and multivariate logistic regression analysis was done and the estimation of odds ratio was applied to establish association between Prakriti and parameters.

## 3 Results

### 3.1 Stability assessment

All recruited CAD patients had a mean ejection fraction score of 47 % and overall quality of life score in SAQ was >70. The data were equally distributed in three *Prakriti* stratified groups (Table 1). Data of three participants were not included in the analysis as they were of mixed *Prakriti (VPK)* type.

**Table 1:** Stability assessment parameters.

Parameters	CAD patients (n = 197)	*Vata* predominant (n = 76)	*Pitta* predominant (n = 42)	*Kapha* predominant (n = 79)	p value
Ejection fraction (%)	47.3 ± 10.9	46.1 ± 10.73	50.3 ± 8.42	46.9 ± 12.08	0.15
Overall quality of life score in SAQ	73.9 ± 13.5	72.3 ± 13.56	74.2 ± 14.52	74.2 ± 14.52	0.70

Values are reported as Mean ± SD, SAQ Seattle angina questionnaire *p*-value compared using One way ANOVA at 5% level of significance.

### 3.2 Prakriti assessment

Overall, in CAD patients *Kapha* predominant *Prakriti* group constituted 39.5 %, followed by *Vata* constituting 38 % and *Pitta* representing 21 %, while the healthy controls, 42 % were of *Vata* predominant *Prakriti,* 28 % of *Pitta* and 29 % of *Kapha* predominant *Prakriti* types (Table 2)*. Sannipattaja (mixed)* type *Prakriti* group comprised a small number of participants including 3 CAD patients and 1 healthy control. Therefore, this group was excluded during analysis.

**Table 2:** *Prakriti* wise stratification in subgroups with % in each group.

Predominant *Prakriti*	CAD patients (n = 200)	Healthy controls (n = 100)
*Vata* participants	76 (38 %)	42 (42 %)
*Pitta* participants	42 (21 %)	28 (28 %)
*Kapha* participants	79 (39.5 %)	29 (29 %)
*Sannipattaja (Mix)* participants	3 (1.5 %)	1 (1 %)

### 3.3 Demographic parameters

The CAD patient group showed male predominance. The mean age of patients in group I was 58.1 ± 10.6 years while it was 37.7 ± 12.0 years in the healthy controls. The literacy rate was poor in CAD patients in all the *Prakriti* stratified groups. The number of participants who smoked/consumed alcohol were more in the CAD patients’ group as compared to healthy controls. No significant differences were observed in dietary habits and BMI in both the groups. Similar trend was observed for most parameters in all *Prakriti* stratified groups (Table 3).

**Table 3:** Demographic and clinical parameters.

Parameters	CAD patients (n = 197)	Healthy controls (n = 99)	*p* value	*Vata* patients (n = 76)	*Vata c*ontrols (n = 42)	*p* value	*Pitta* patients (n = 42)	*Pitta* controls (n = 28)	*p* value	*Kapha* patients (n = 79)	*Kapha* controls (n = 29)	*p* value
**Demographic parameters**												
Age (in years)	58.1 ± 10.6	37.7 ± 12.0	<0.001	60.9 ± 10.7	39.1 ± 12.8	<0.001	55.3 ± 10.5	36.1 ± 10.6	<0.001	56.8 ± 10.2	37.2 ± 12.3	<0.001
Gender Male	175 (88.8 %)	47 (48.0 %)	<0.001	68 (89.5 %)	21 (50 %)	<0.001	37 (88.1 %)	14 (50 %)	0.001	70 (88.6 %)	13 (42.9 %)	<0.001
Female	22 (11.2 %)	52 (52.0 %)		8 (10.5 %)	21 (50 %)		5 (11.9 %)	14 (50 %)		9 (11.4 %)	16 (57.1 %)	
Married	195 (99.0 %)	58 (58.8 %)	<0.001	74 (97.4 %)	28 (65.1 %)	<0.001	42 (100.0 %)	15 (52.9 %)	<0.001	79 (100.0 %)	15 (53.6 %)	<0.001
Unmarried	2 (1.0 %)	41 (41.2 %)		2 (2.6 %)	14 (34.9 %)		0 (0.0 %)	13 (47.1 %)		0 (0.0 %)	14 (46.4 %)	
Illiterate	17 (8.6 %)	0 (0.0 %)	<0.001	10 (13.2 %)	0 (0.0 %)	<0.001	2 (4.8 %)	0 (0.0 %)	0.017	5 (6.3 %)	0 (0.0 %)	0.016
Literate	36 (18.3 %)	3 (0.3 %)		18 (23.7 %)	0 (0.0 %)		7 (16.7 %)	2 (7.4 %)		11 (13.9 %)	0 (0.0 %)	
High school	73 (37.1 %)	16 (16.3 %)		26 (34.2 %)	5 (11.6 %)		18 (42.9 %)	4 (14.8 %)		29 (36.7 %)	7 (25.0 %)	
Graduate	53 (26.9 %)	53 (54.1 %)		16 (21.1 %)	25 (58.1 %)		11 (26.2 %)	15 (55.6 %)		26 (32.9 %)	13 (46.4 %)	
Professional course	18 (9.1 %)	27 (27.6 %)		6 (7.9 %)	12 (30.2 %)		4 (9.5 %)	6 (22.2 %)		8 (10.1 %)	9 (28.6 %)	
Vegetarian	51 (25.9 %)	35 (36.7 %)	0.139	18 (23.7 %)	12 (30.2 %)	0.44	9 (21.4 %)	9 (33.3 %)	0.497	24 (30.4 %)	19 (52.0 %)	0.121
Vegan	4 (2.0 %)	1 (1.0 %)		2 (2.6 %)	0 (0.0 %)		1 (2.4 %)	1 (3.7 %)		1 (1.3 %)	0 (0.0 %)	
Mixed	142 (72.1 %)	63 (30.0 %)		56 (73.7 %)	30 (69.8 %)		32 (76.2 %)	17 (63.0 %)		54 (68.4 %)	14 (48.0 %)	
Addiction-yes	145 (73.6 %)	40 (40.8 %)	<0.001	56 (73.7 %)	20 (48.8 %)	0.006	30 (71.4 %)	10 (37.0 %)	0.005	59 (74.7 %)	9 (32.1 %)	<0.001
No	52 (26.4 %)	59 (59.2 %)		20 (26.3 %)	22 (51.2 %)		12 (28.6 %)	18 (63.0 %)		20 (25.3 %)	20 (67.9 %)	
**Clinical parameters**												
Systolic blood pressure (mmHg)	124.9 ± 14.8	120.0 ± 12.5	0.005	123.7 ± 16.4	122.7 ± 13.2	0.72	124.5 ± 12.3	117.6 ± 12.5	0.028	126.2 ± 14.5	118.2 ± 10.9	0.009
Diastolic blood pressure (mmHg)	80.3 ± 8.0	78.4 ± 6.9	0.052	79.0 ± 8.90	78.7 ± 7.56	0.85	79.9 ± 7.4	78.6 ± 7.1	0.474	81.7 ± 7.3	77.8 ± 5.7	0.012
Body mass index (Kg/m^2^)	25.3 ± 3.3	25.9 ± 4.1	0.189	24.2 ± 3.5	25.2 ± 4.1	0.155	25.3 ± 2.9	25.4 ± 3.0	0.905	26.5 ± 2.9	27.6 ± 4.6	0.147

Values are presented as Mean ± SD or median (IQR) in parameters with high standard deviation.

### 3.4 Biochemical parameters and biomarkers

Parameters like TLC, fasting blood sugar, urea, creatinine, uric acid, HbA1c, MDA, NT-pro BNP were significantly high in CAD patients as compared to controls. However, lymphocyte count, total cholesterol, low density lipoproteins and high-density lipoproteins were significantly low in CAD patients as compared to controls. Further, comparison of CAD patients and controls in stratified groups of *Prakriti* showed total lymphocyte count to be significantly high in *Vata* and *Kapha* CAD patients when compared to healthy controls, while, there was no significant difference in the *Pitta* group. Neutrophils were significantly higher in *Vata* and *Pitta* CAD patients. Leucocytes were significantly low in all the three *Prakriti* of CAD patients. Monocytes were significantly low in *Pitta* patients. Platelet count was significantly less in *Vata* and *Kapha* patients, but there was no change in the *Pitta* group. Fasting blood sugar was significantly high in *Kapha* patients. Alkaline phosphatase was significantly high in *Pitta* patients. Urea was significantly high in all the three *Prakriti* subtypes of CAD patients. Creatinine and uric acid were significantly high in *Vata* and *Kapha* patients. Total cholesterol and LDL were significantly higher in all the three *Prakritis* of the healthy controls. HDL was significantly high in *Vata* controls. HbA1c was high in CAD patients of all three *Prakritis*. However, most of the biochemical parameters, apart from fasting blood sugar and HbA1c, were within the normal range.

IL-6 was significantly high in *Vata* patients as compared to healthy controls. NT-pro BNP was significantly high in all three stratified groups of patients when compared with controls. MDA was significantly high in *Kapha* patients only (Table 4) Further, data of each *Prakriti* type of CAD patients were compared with all healthy controls. This analysis involved comparison *Vata, Pitta* and *Kapha Prakriti* patients with healthy controls of all *Prakriti*. Results demonstrated that the catalase was lowest and PCSK9 levels were highest in *Kapha* patients when compared with heathy controls. Serum albumin, PCSK9 and Lp- PLA2 were lowest and IL-6 highest in *Vata* patients in comparison with healthy controls. NT-pro BNP and alkaline phosphatase were higher in *Pitta* CAD patients as compared. Data analysis between overall patients and controls, further in each *Prakriti* stratified group with all healthy controls has been presented in Tables 4.

**Table 4:** Hematological parameters, Liver function test, Kidney function test, blood sugar fasting and HbA1c, Lipid profile, Cardiac-Biomarkers in overall and specific *Prakriti* patients of CAD.

Parameters	CAD patients (197)	All healthy controls (n = 99)	p value overall	*Vata* CAD patients (n = 77)	p value *Vata* CAD patients verses all controls	*Pitta* CAD patients (n = 41)	p value *Pitta* CAD patients verse all controls	*Kapha* CAD patients (n = 79)	p value *Kapha* CAD patients verses all controls
**Hematological parameters in overall and specific *Prakriti* patients of CAD**									
Haemoglobin (g/dL)	13.4 ± 1.7	13.7 ± 1.8	0.261	13.2 ± 1.65	0.191	13.5 ± 1.75	0.449	13.6 ± 1.8	0.345
Total leucocyte counts 10^3^/μL	8.0 ± 2.2	6.8 ± 1.7	<0.001	8.3 ± 2.2	0	7.96 ± 2.65	0.005	7.8 ± 1.7	0
Neutrophils%	58 ± 0.1	54 ± 9	<0.001	59 ± 9	0	58 ± 9	0.002	57 ± 8	0.006
Lymphocytes%	29 ± 0.1	35 ± 9	<0.001	29 ± 9.9	0	28 ± 9	0	0.3 ± 7	0
Monocytes%	11 ± 0.1	16 ± 1.0	0.544	15 ± 0.7	0.979	8 ± 0.1	0.591	8 ± 0.1	0.499
Platelet count (10^3^/μL)	212.81 ± 81.8	269.7 ± 76.4	<0.001	199.4 ± 64.6	0	227.4 ± 92.1	0.004	218 ± 89.6	0
ESR (mm)	23.9 ± 4.5	22.1 ± 12.9	0.298	23.5 ± 15.4	0.874	25.6 ± 15.3	0.534	23.4 ± 13.1	0.517
Monocytes lymphocyte ratio	29.2 (21.1, 36.2)	19.5 (12.1, 25.5)	<0.001	30 (27.1, 35.6)	0.033	28.8 (12.1, 38.8)	0.1	32.4 (9.8, 43.5)	0.926
**Liver function test in overall and specific *Prakriti* patients of CAD**									
Total protein (g/dL)	7.6 ± 0.7	7.7 ± 0.6	0.174	7.4 ± 0.8	0.006	7.6 ± 0.5	0.257	7.8 ± 0.7	0.762
S. albumin (g/dL)	4.6 ± 0.5	4.6 ± 0.4	0.633	4.5 ± 0.6	0.008	4.7 ± 0.4	0.57	4.7 ± 0.4	0.263
S. globulin (g/dL)	3.0 ± 0.5	3.0 ± 0.5	0.931	3.0 ± 0.5	0.919	2.9 ± 0.5	0.729	3.1 ± 0.59	0.957
S. bilirubin (mg/dL)	0.7 ± 0.1	0.6 ± 0.9	0.766	0.7 ± 0.5	0.418	0.6 ± 0.5	0.902	0.7 ± 0.4	0.863
AST (IU/L)	24.0 (20.0, 31.5)	24.0 (17.0, 31.25)	0.997	25.0 (20.0, 34.0)	0.215	24.5 (19.8, 28.0)	0.705	25.0 (16.0, 33.0)	0.628
ALT (IU/L)	25.0 (19.0, 33.5)	19.7 (10.5, 24.3)	0.862	30.3 (11.6,43.2)	<0.001	31.4 (24.3,43.5)	<0.001	32.4 (0.2,0.45)	<0.001
S. alkaline phosphatase (IU/L)	105.1 ± 36.7	97.2 ± 33.3	0.07	104.9 ± 34.4	0.118	117.9 ± 48.5	0.008	98.4 ± 28.2	0.827
**Kidney function test, blood sugar fasting and HbA1c in overall and specific *Prakriti* patients of CAD**									
Blood urea (mg/dl)	27.4 ± 11.5	21.4 ± 6.8	<0.001	29 ± 14	0	25.5 ± 10.2	0.024	26.7 ± 9.3	0
Creatinine (mg%)	0.9 ± 0.3	0.7 ± 0.2	<0.001	1.0 ± 0.3	0	0.8 ± 0.2	0.007	0.9 ± 0.2	0
Uric acid (mg/dL)	5.8 ± 1.7	5.0 ± 1.4	<0.001	6.1 ± 1.9	0	5.2 ± 1.4	0.258	5.7 ± 1.5	0
Fasting blood sugar (mg/dL)	117.4 ± 28	95.6 ± 31.8	<0.001	116.2 ± 54.9	0.002	116 ± 46.8	0.001	129.3 ± 41.7	0
HbA1c (%)	6.8 ± 1.6	5.8 ± 1.0	<0.001	6.7 ± 1.7	0	6.6 ± 1.3	0	7.1 ± 1.5	0
**Lipid profile in overall and specific *Prakriti* patients of CAD**									
Total cholesterol (mg/dL)	136.2 ± 44	184.0 ± 40.3	<0.001	137.5 ± 44.2	0	127.9 ± 32.2	0	140.6 ± 46.8	0
LDL (mg/dL)	65.8 ± 34.2	114.8 ± 36.9	<0.001	67.7 ± 32.1	0	58.7 ± 25.3	0	67.8 ± 39.6	0
HDL (mg/dL)	42.2 ± 11.5	48.4 ± 13.8	<0.001	41.7 ± 10.6	0	41.8 ± 13	0.002	42.9 ± 11.6	0.004
**Cardiac biomarkers in overall and specific *Prakriti* patients of CAD**									
IL-6 (pg/mL)	5.3 (2.9, 13.2)	4.8 (2.8, 8.2)	0.819	8.7 (4.7, 16.1)	0	5.5 (2.7, 13)	0.409	4 (1.9, 7.3)	0.207
PCSK9 (ng/mL)	93.1 (54.6, 216.2)	116.7 (58.7, 183.7)	0.24	74.1 (42.7, 156.5)	0.042	127 (55.1, 235.4)	0.756	141.3 (63.8, 246.3)	0.013
LpPLA2 (ng/mL)	152.9 (79.1, 200.1)	165.9 (80.5, 235.4)	0.596	110.9 (7.0, 199.4)	0.004	147.7 (103.7, 191)	0.494	173.8 (120.9, 206.1)	0.902
CRP (mg/L)	26.2 (21.4, 42.6)	26.6 (16.2, 43.1)	0.787	26.4 (16.6, 46.3)	0.732	25.65 (20.43, 38.94)	0.548	28.6 (21.4, 42.9)	0.493
NT-pro BNP (pg/mL)	248.5 (83.3, 497.3)	26.0 (20.0, 71.0)	<0.001	247 (107, 517.8)	0	376 (63.5, 711.5)	0	223 (80, 464)	0
GSH (nmol/ml)	25 (13.6, 31.8)	24.4 (10.5, 32.7)	0.882	27.3 (17.5, 32.8)	0.141	23.6 (8.8, 31.8)	0.752	22.2 (13.4, 30.7)	0.746
MDA (nmol/ml)	7.3 (3.8, 10.8)	5.2 (2.3, 8.1)	0.002	7.2 (4.9, 9.2)	0.04	6.7 (3.5, 10)	0.089	9.4 (3.8, 12.1)	0.004
Extra cellular SOD (mg/dL)	78.3 (54.0, 120.1)	87.6 (64.1, 134.7)	0.239	75.4 (59.0, 112)	0.326	78.3 (54, 153.7)	0.484	86.9 (36.5, 127)	0.35
Catalase (μM)	74.1 (68.6, 84.1)	76.7 (67.5, 156.8)	0.051	75.6 (71.9, 85.6)	0.536	72.5 (69, 89.2)	0.234	68.3 (21.6, 85.3)	0.01

Further, the concise data showing only the significant results in different *Prakriti* groups and within *Prakriti* groups has been presented in Table 5.

**Table 5:** Parameters with significant differences amongst the groups.

Parameters	Over all CAD patients and healthy controls	*Vata* CAD patients and healthy controls	*Pitta* CAD patients and healthy controls	*Kapha* CAD patients and healthy controls	*Within VPK CAD patients p value*
Total leucocyte counts	↑↑	↑↑∗	↑↑ #	↑↑$	0.321
Neutrophils	↑↑	↑↑∗	↑↑#	No Difference	0.435
Lymphocytes	↓↓	↓↓∗	↓↓#	↓↓$	0.775
Platelet count	↓↓	↓↓∗	No Difference #	↓↓ $	0.05
Monocytes lymphocyte ratio	↑↑	↑↑∗	No Difference	No Difference	0.526
Total cholesterol	↓↓	↓↓∗	↓↓#	↓↓	0.002
LDL	↓↓	↓↓∗	↓↓#	↓↓	0.04
HDL	↓↓	↓↓∗	No Difference	No Difference	0.306
Blood urea	↑↑	↑↑∗	↑↑#	↑↑$	0.432
Creatinine	↑↑	↑↑∗	No Difference#	↑↑$	0.018
Uric acid	↑↑	↑↑∗	No Difference	↑↑$	0.236
Fasting blood sugar	↑↑	No Difference ∗	No Difference #	↑↑ $	0.899
HbA1c	↑↑	↑↑∗	↑↑#	↑↑$	0.325
S. alkaline phosphatase	No Difference	No Difference	↑↑#	No Difference	0.028
IL-6	No Difference	No Difference∗	No Difference	No Difference	0.001
PCSK9	No Difference	No Difference∗	No Difference	No Difference$	0.008
LpPLA2	No Difference	Difference∗	No Difference	No Difference	0.007
NT-pro BNP	↑↑	↑↑∗	↑↑#	↑↑$	0.047
MDA	↑↑	No Difference∗	No Difference	↑↑$	0.129
Catalase	No Difference	No Difference	↑↑	No Difference $	0.062

∗/#/$ shows significant difference in specific Prakriti classified groups (V/P/K) compared to all healthy controls.

### 3.5 Drug therapy in all patients

A detailed information regarding the drug therapy of each patient was documented and analysis was done with the percentage of the individual drug prescribed in three stratified groups of patients. There was no significant difference in three predominant *Prakriti* groups. The details of the drugs prescribed to the patients overall and further in *Prakriti* stratified groups have been mentioned in Table 6.

**Table 6:** Drug therapy records in overall and *Prakriti* classified groups of CAD patients.

S. No.	Types of drug therapy	Percentage of patients (n = 197)	% Vata CAD patients (n = 76)	% Pitta CAD patients (n = 42)	% Kapha CAD patients (n = 79)	p value
Standard of care medicines for CAD						
**1**	Angiotensin-converting enzyme inhibitors	54.3 %	56.6 %	50 %	54.4 %	0.472
**2**	Angiotensin receptor blockers	39.6 %	39.5 %	42.9 %	38.0 %	0.251
**3**	Calcium channel blockers	29.9 %	26.3 %	28.6 %	34.2 %	0.552
**4**	Beta blockers	67.5 %	71.1 %	66.7 %	64.6 %	0.762
**5**	Diuretics	35 %	31.6 %	31.0 %	40.5 %	0.174
**6**	Statins	88.3 %	88.2 %	83.3 %	91.1 %	0.162
**7**	Anti-inflammatory/Anti-platelet agent (Most commonly prescribed ecosprin)	58.9 %	64.5 %	54.8 %	58.2 %	1.227
**8**	Antiplatelets: Clopidogrel, ticagrelor, prasugrel, and dipyridamole	72.6 %	72.4 %	76.2 %	70.9 %	0.395
**9**	Vasodilators	14.2 %	14.5 %	14.3 %	13.9 %	0.982
**10**	Nitrates	47.7 %	46.1 %	47.6 %	49.4 %	0.178
**11**	HCN channel blockers (Ivabradine)/ARNI Angiotensin Receptor-Neprilysin Inhibitor	14 %	14 %	13.2 %	18.8 %	0.127
**Medicines other than** s**tandard of** c**are for CAD**						
**11**	Proton pump inhibitors	44.7 %	40.8 %	54.8 %	46 %	0.228
**12**	Anti diabetics	31.5 %	29 %	28.6 %	35.4 %	0.996
**13**	Anti tubercular therapy/Thyroid agents/calcium/multivitamin/vit-D/iron	24 %	24.2 %	21.2 %	22.6 %	0.926

### 3.6 Univariate and multivariate logistic regression analysis

On univariate and multivariate logistic regression analysis among the variables, gender and age were found to be significantly associated with the risk of CAD. The odds of having CAD were higher in males as compared to females [AOR (95 % CI): 8.9 (2.8–28.7)]. The odds were also higher in those above the age of 50 years as compared to those below this age [AOR (95 % CI): 6.2 (1.8–21.7)].

Among the biochemical parameters NT-pro BNP was found to be significantly associated with risk of developing CAD. The risk of getting the disease was higher in individuals having NT-pro BNP levels >125 ng/ml as compared to those with below that level [AOR (95 % CI): 21.6 (5.3–86.9)].

To establish the risk between *Prakriti* types, the parameters in which significant difference was observed were included for analysis and differences in risk associations in different *Prakriti* types were observed. The multivariate logistic regression also revealed a significant association between *Prakriti* types and CAD. The likelihood of having CAD after adjusting for age, gender, literacy status, addictions and other biochemical parameters was higher in individuals having *Kapha Prakriti* as compared to *Vata Prakriti* [AOR (95 % CI): 4.1 (1.2–14.1)] (Table 7, Table 8).

**Table 7:** Univariate and multivariate logistic regression analysis between CAD patients and healthy controls using different variables.

		CAD patients	Controls	COR (95 % CI)	AOR (95 % CI)
*Prakriti*	*Pitta*	44 (65.7 %)	23 (34.3 %)	1.2 (0.6–2.2)	1.6 (0.4–6.8)
	*Kapha*	78 (72.2 %)	30 (27.8 %)	1.6 (0.9–2.8)	4.1 (1.2–14.1)
	*Vata*	75 (62.0 %)	46 (38.0 %)	Ref	Ref
Gender	Male	161 (87.5 %)	23 (12.5 %)	14.8 (8.2–26.7)	8.9 (2.8–28.7)
	Female	36 (32.1 %)	76 (67.9 %)	Ref	Ref
Age	**>** 50 years	157 (75.8 %)	50 (24.2 %)	3.9 (2.3–6.5)	6.2 (1.8–21.7)
	**≤** 50 years	40 (44.9 %)	49 (55.1 %)	Ref	Ref
Illiterate	Able to read and write	53 (96.4 %)	2 (3.6 %)	17.9 (4.3–74.9)	9.2 (0.97–87.9)
	Educated	144 (59.8 %)	97 (40.2 %)	Ref	Ref
Addiction	Yes	138 (88.5 %)	18 (11.5 %)	10.5 (5.8–19.1)	31.5 (5.5–180.9)
	No	59 (42.1 %)	81 (57.9 %)	Ref	Ref
Blood sugar fasting	>110 mg/dL	89 (89.0 %)	11 (11.0 %)	6.7 (3.4–13.2)	8.4 (1.8–38.4)
	**≤** 110 mg/dL	107 (54.9 %)	88 (45.1 %)	Ref	Ref
Serum Alkaline Phosphatase	>147 IU	17 (81.0 %)	4 (19.0 %)	2.2 (0.7–6.8)	2.6 (0.2–27.9)
	**≤** 147 IU	180 (65.5 %)	95 (34.5 %)	Ref	Ref
Uric Acid	>7.4 mg%	29 (78.4 %)	8 (21.6 %)	0.5 (0.2–1.2)	2.3 (0.4–15.8)
	≤7.4 mg%	168 (64.9 %)	91 (35.1 %)	Ref	Ref
Total Cholesterol	>200 mg/dL	19 (32.8 %)	39 (67.2 %)	0.2 (0.1–0.3)	0.8 (0.2–4.1)
	**≤** 200 mg/dL	178 (74.8 %)	60 (25.2 %)	Ref	Ref
LDL	>100 mg/dL	21 (26.3 %)	59 (73.7 %)	0.0 (0.04–0.2)	0.1 (0.03–0.5)
	**≤** 100 mg/dL	176 (81.5 %)	40 (18.5 %)	Ref	Ref
HDL	**<** 40 mg/dL	94 (83.9 %)	18 (16.1 %)	4.1 (2.3–7.4)	2.7 (0.8–9.5)
	≥40 mg/dL	103 (56.0 %)	81 (44.0 %)	Ref	Ref
HbA1c	**>** 6.5 %	100 (84.7 %)	18 (15.3 %)	4.7 (2.6–8.3)	1.2 (0.3–4.5)
	**≤** 6.5 %	97 (54.5 %)	81 (45.5 %)	Ref	Ref
IL-6	**>** 5 pg/ml	101 (74.8 %)	34 (25.2 %)	2.0 (1.2–3.3)	2.1 (0.7–6.3)
	**≤** 5 pg/ml	96 (59.6 %)	65 (40.4 %)	Ref	Ref
PCSK9	**>** 300 ng/ml	26 (76.5 %)	8 (23.5 %)	1.7 (0.8–3.9)	0.7 (0.2–3.2)
LpPLA2	**≤** 300 ng/ml	171 (65.3 %)	91 (34.7 %)	Ref	Ref
	**>** 40 ng/ml	152 (65.8 %)	79 (34.2 %)	0.9 (0.5–1.6)	0.7 (0.2–2.8)
	**≤** 40 ng/ml	45 (69.2 %)	20 (30.8 %)	Ref	Ref
NT-pro BNP	>125 pg/ml Age < 75	124 (93.9 %)	8 (6.1 %)	19.3 (8.9–42.1)	21.6 (5.4–86.9)
	**≤** 125 pg/ml Age < 75	73 (44.5 %)	91 (55.5 %)	Ref	Ref

COR = Crude Odds Ratio, CI = Confidence Interval, AOR = Adjusted Odds Ratio.

**Table 8:** Adjusted odds-ratio for specific *Prakriti* with other risk factors among stratified CAD patients and healthy controls.

Risk factors (adjusted with)	*Prakriti* (Adjusted Odds-ratio with 95 % Confidence interval)		
	*Pitta*	*Kapha*	*Vata*
Gender	0.92 (0.43–1.98)	1.44 (0.74–2.84)	Ref
Age	1.41 (0.73–2.74)	1.76 (0.98–3.17)	Ref
Literacy	1.27 (0.66–2.47)	1.95 (1.1–3.50)	Ref
Smoking	1.32 (0.69–2.53)	1.61 (0.90–2.87)	Ref
Addiction	1.32 (0.64–2.71)	1.81 (0.95–3.44)	Ref
Blood sugar fasting levels	1.08 (0.55–2.1)	1.46 (0.81–2.65)	Ref
Serum Alkaline Phosphatase levels	1.21 (0.64–2.26)	1.70 (0.97–3.0)	Ref
Uric acid levels	1.23 (0.66–2.31)	1.62 (0.93–2.85)	Ref
Cholesterol levels	1.11 (0.57–2.17)	16 (0.88–2.1)	Ref
LDL levels	1.11 (0.53–2.3)	1.72 (0.89–3.31)	Ref
HDL levels	1.1 (0.57–2.12)	1.55 (0.87–2.77)	Ref
HbA1c levels	1.05 (0.54–2.02)	1.38 (0.77–2.48)	Ref
IL-6 levels	1.33 (0.70–2.52)	1.91 (1.07–3.41)	Ref
PCSK9 levels	1.16 (0.62–2.16)	1.52 (0.87–2.68)	Ref
LpPLA2 levels	1.25 (0.66–2.36)	1.72 (0.96–3.05)	Ref
NT-pro BNP levels	1.05 (0.49–2.23)	1.95 (1.02–3.74)	Ref

## 4 Discussion

CAD is the one of the most common causes of mortality [4] and is due to different etiologies [45]. Oxidative stress plays a key role at distinct pathophysiological phases and severity of the disease [46]. It can intensify inflammation and may lead to low-density lipoprotein cholesterol oxidation and endothelial dysfunctions. At the same time stress at cellular levels may change the proportions of oxidative stress molecules and antioxidants.

In the present study, male predominance was observed in CAD patients as compared to controls. This could be because of increased number of smokers amongst males, leading to hypertension. Moreover, they are deprived of protective HDL cholesterol as compared to females resulting in increased gender ratio of prevalence as well as mortality due to CAD in males [47]. The incidence of the coronary artery calcification is reported to be more in the middle aged and elderlies [48] which could have contributed to the higher age group of patients observed in our study. Overall literacy level was poor in patients. This agrees with the findings of an earlier study wherein an inverse association of literacy status with all-cause mortality due to CAD was observed in Indians [49]. Furthermore, there was a higher prevalence of individuals who were consuming cigarettes or tobacco in the CAD patient group. The detrimental impact of cigarettes and nicotine on vascular health are well documented [50]. Smoking heightens sympathetic activity and triggers vasospasm, potentially leading to myocardial necrosis. Nicotine has been demonstrated to induce damage to the coronary vascular endothelium and causes ischemic necrosis/toxic necrosis. Moreover, nicotine is known to contribute to calcium deposition, fostering atheroma formation and leading to CAD [50].

In demographic analysis, it was observed that percentage of CAD patients was high in *Kapha* group while in healthy controls, there was predominance of *Vata Prakriti*. Regression analysis demonstrated that the *Kapha Prakriti* patients were more inclined towards risk for the predisposition of CAD. Our observations are in coherence with the findings of a previous study wherein the prevalence of CVD was more in *Kapha Prakriti* individuals [23]. Ayurveda texts have also demonstrated an increased susceptibility of *Kapha* individuals towards cardiovascular diseases with specific reference to atherosclerosis (CAD) [23]. Some of the reasons could be the involvement of factors like poor sugar control and high PCSK9 indirectly disturbing liver metabolism in *Kapha Prakriti*, as observed in the present study, making *Kapha Prakriti* individuals comparatively more susceptible towards CAD predisposition. However, no differences in the dietary habits and BMI were observed amongst the participants in both the groups and in *Prakriti* stratified groups as well.

In this study, we have assessed biochemical parameters/biomarkers in *Prakriti* stratified CAD patients and healthy controls. We hypothesized that *Prakriti* stratification of CAD patients/healthy controls and comparison of their parameters could help in identifying specific variations in different *Prakriti* types. Changes in hematological parameters have been reported to affect the prognosis of CAD [51]. Almost all subtypes of white blood cells are known to be increased in CAD [52]. A study has postulated the association of high neutrophil, eosinophil, lymphocyte, monocyte, or basophil count in angiographically proven CAD [53]. WBCs possess the ability to combine and embolize to microvascular sites [54]. Adherence to the microvascular site results in inflammation and vascular injury which increases oxidative stress by the production of superoxide radicals, proteolytic enzymes and arachidonic acid metabolites [55]. The same was reflected in our study patients and all stratified groups. A higher platelet count may be responsible for acute ischemic condition [46], nevertheless in the present study, the platelet count was lower in the patient group. This could be due to our stable CAD patients receiving anti-platelet drug therapy as a part of their treatment regimen. Although, the apparent platelet function depends on the platelet reactivity and not on the count, nonetheless, studies have shown a decrease in total platelet count in stable CAD patients on standard drug (antiplatelet) therapy [56]. Fasting blood sugar and HbA1c was higher in all CAD patients. However, the fasting blood sugar levels were significantly higher in patients with *Kapha* predominant type *Prakriti* as compared to controls, while in *Vata* and *Pitta* groups sugar levels were not significantly different. This raised blood sugar and HbA1c observed in our study is in accordance with have reports diabetic patients were shown to have an increased propensity to develop CAD [57,58].

In our study population, vitamin D levels were retrieved from their biochemical records. Vitamin D deficiency was more prevalent in healthy controls when compared to the CAD patients; probably due to the vitamin D supplementation with the standard of care during CAD management in the patients. *Prakriti* classification showed higher levels of vitamin D deficiency in *Pitta* patients and *Kapha* controls. IL-6 was significantly higher only in *Vata* patients. IL-6 has also been shown as a predictor of increased oxidative stress [59]. Creatinine and uric acid were increased in CAD patients and *Prakriti* stratification showed increased levels in *Vata* and *Kapha* patients. MDA was higher in CAD patients compared to controls and *Prakriti* classification showed significantly higher levels only in *Kapha* patients. An increase in level of free radicals due to oxidative stress causes increased production of MDA [60]. The observations of our study concur with previous studies reporting differences in biochemical parameters in people of different constitution types. Further *Prakriti* stratification could reflect that *Pitta* predominant *Prakriti* participants were least disturbed in terms of their platelet count, serum creatinine, uric acid, and HDL. However, *Pitta* controls had higher catalase values. Higher catalase has been reported to increase the breakdown of hydrogen peroxide to produce water and oxygen; thus, resulting in a decrease in oxidative stress [61].

Previous studies have exhibited differences in biochemical parameters in different *Prakriti* individuals which correlated with their genetic profile. For instance, increased LDL, reduced prothrombin and low expression of genes related to fibrinolysis (KRT1 and F2) have been reported in *Kapha* individuals, thus predisposing this *Prakriti* individuals to atherosclerotic conditions [35]. Further, a link between Egl-9 Family Hypoxia Inducible Factor 1 (EGLN1) and von Willebrand factor (vWF) in a constitution specific manner (higher in *Kapha Prakriti*) have been demonstrated [62]. In a recent study, exome sequencing of healthy individuals of extreme constitutions also provided leads towards disease predisposition [63]. In the present study, we have selected predominant type of *Prakriti* in contrast to most previous studies wherein extreme *Prakriti* types were selected. Although the *Prakriti* segregated patients in the three *Prakriti* groups were uniformly distributed in terms of their angina/echocardiography score, still we could find some differences in the pathways for their disease predisposition. This finding is of great importance and could be applied in the personalized management of CAD.

Further, when CAD patients of specific *Prakriti* were compared with the healthy controls, the findings obtained were more precise. For instance, PCSK9 levels and catalase were higher in *Kapha* patients. Recent evidence showed regulatory effect of PCSK9 on redox system, which may be the predisposing factor among *Kapha* individuals [64]. On the contrary, PCSK9 was comparatively low in *Vata* patients while IL-6 levels were high, indicating an involvement of the immune and inflammatory pathways for disease predisposition in *Vata* patients. Previous studies have also given lead towards getting early readouts of disease predisposition even in healthy individuals when they were phenotypically *Prakriti* classified [35]. Additionally, a research study provided validation for the significance of *Prakriti*-based classification in evaluating the distinct responses of autonomic cardiac modulation in different postures among healthy individuals [65].

The present study is of great importance as it could give insights on how different *Prakriti* individuals display differential susceptibility towards the parameters performed in the study. Therefore, the approach of segregating the individuals with phenotypic differences may provide immense help in identifying individuals with higher predisposition to CAD. Moreover, response towards the same medications can be different in different individuals. This forms the basis of pharmacogenomics and can also be applied for the management of CAD [66]. Efforts have been made to validate the concept of *Prakriti* in *Ayurveda* with artificial intelligence through the use of a powerful dense neural network deep learning algorithms for predicting *Prakriti* types which may reduce bias of only being subjective analysis [67].

In the present study, the segregation of drug therapy from overall CAD patients to *Prakriti* stratified groups demonstrated no significant difference but while the overall *Vata Prakriti* patients had the lowest platelet count as compared to patients of other *Prakritis*, which may be indication for requirement of lower doses of antiplatelet drugs in *Pitta Prakriti* patients. Further, low PCSK9 levels in *Vata* patients and high NT-pro BNP in *Pitta* patients observed in our study may also provide a lead towards pharmacogenomics-based therapy with *Prakriti* evaluation. Although there were no differences in drug therapies in the three *Prakriti* groups, differences in the level of various markers were observed, which may affect the pharmacotherapy. Thus, *Prakriti* classification will be a valuable approach in the era of personalized medicine.

## 5 Conclusion

The available guidelines for CAD management emphasize on the early diagnosis and timely management. Even after diagnosis and treatment, the patients need to be carefully monitored throughout their lifespan. In our study, the stable CAD patients fared significantly worse in terms of their total leucocyte count, NT-pro BNP and HbA1c. This reflects inflammatory stress, myocardial injury, and poor glycemic control. NT-pro BNP was observed as a good biomarker for severity assessment in CAD patients and therefore, this may be considered for periodic evaluation. Hence, clinical, and biochemical assessments at regular intervals may be recommended. Comparison of CAD patients and healthy controls did not show significant difference in several parameters. However, *Prakriti* assessment revealed differences in those parameters. Therefore, *Prakriti* identification may facilitate categorizing individuals predisposed to CAD based on the susceptible pathways involved. These noted variations may aid in providing guidance to modify the treatment strategies. The present study was an attempt to integrate knowledge of traditional ayurvedic concepts with the contemporary science. To conclude, several biochemical parameters/biomarkers were suggested as risks for CAD and *Prakriti* classification provided more insights of the precise risks. Patients with *Kapha Prakriti* are more likely to be at risk for the disease*.* Hence, implementing a dual approach of incorporating *Prakriti* assessment along with estimations of biochemical parameters/biomarkers could potentially help in personalized management of CAD.

## Author contribution

PD: conceptualization, designing, conducting, data acquisition, data analysis, interpretation, writing-original draft preparation, writing-reviewing, and editing. BP: designing, execution, analysis, interpretation, and writing-reviewing and editing. SS: designing, data acquisition, writing-reviewing, and editing. SP: data analysis. SKM: writing-reviewing and editing. KH R: designing, analysis, interpretation, writing-reviewing, and editing. All authors read and approved the final manuscript.

## Ethical statement

Written approval from the Institute ethics committee (IEC510/05/10,2018, RP-40/2018) was obtained before commencement of the study. All participants provided written informed consent.

## Declaration of generative AI in scientific writing

The authors declare that this article did not use generative AI or AI-assisted technologies.

## Conflict of interest

The authors declare that they have no known competing financial interests or personal relationships that could have appeared to influence the work reported in this paper.

## Data Availability

The data will be made available on reasonable request from the first or corresponding author.

## References

[1] Barquera S., Pedroza-Tobías A., Medina C., Hernández-Barrera L., Bibbins-Domingo K., Lozano R. (2015). Global overview of the epidemiology of atherosclerotic cardiovascular disease. Arch Med Res.

[2] Tibaut M., Petrovič D. (2016). Oxidative stress genes, antioxidants and coronary artery disease in type 2 diabetes mellitus Cardiovasc Hematol Agents. Med Chem.

[3] Vasan R.S. (2006). Biomarkers of cardiovascular disease: molecular basis and practical considerations. Circulation.

[4] Lubrano V., Pingitore A., Traghella I., Storti S., Parri S., Berti S. (2019). Emerging biomarkers of oxidative stress in acute and stable coronary artery disease: levels and determinants. Antioxidants (Basel).

[5] Hulsmans M., Van Dooren E., Holvoet P. (2012). Mitochondrial reactive oxygen species and risk of atherosclerosis. Curr Atheroscler Rep.

[6] Hulsmans M., Holvoet P. (2010). The vicious circle between oxidative stress and inflammation in atherosclerosis. J Cell Mol Med.

[7] Libby P., Theroux P. (2005). Pathophysiology of coronary artery disease. Circulation.

[8] Haft J.I. (1979). Role of blood platelets in coronary artery disease. Am J Cardiol.

[9] Guo X., Ma L. (2023). Inflammation in coronary artery disease-clinical implications of novel HDL- cholesterol-related inflammatory parameters as predictors. Coron Artery Dis.

[10] Zaman A.G., Helft G., Worthley S.G., Badimon J.J. (2000). The role of plaque rupture and thrombosis in coronary artery disease. Atherosclerosis.

[11] Zhou M., Yu Y., Luo X., Wang J., Lan X., Liu P. (2021). Myocardial ischemia-reperfusion injury: therapeutics from a mitochondria-centric perspective. Cardiology.

[12] Goldberg L., Mekel J., Landless P., Smith D., Grigorov V. (2001). Myocardial viability- mechanisms of reversible myocardial dysfunction and diagnosis in coronary artery disease. Cardiovasc J South Afr.

[13] Vernon S.T., Tang O., Kim T., Chan A.S., Kott K.A., Park J. (2021). Metabolic signatures in coronary artery disease: results from the bioHEART-CT study. Cells.

[14] Medina-Leyte D.J., Zepeda-García O., Domínguez-Pérez M., González-Garrido A., Villarreal-Molina T., Jacobo-Albavera L. (2021). Endothelial dysfunction, inflammation, and coronary artery disease: potential biomarkers and promising therapeutical approaches. Int J Mol Sci.

[15] Khosravi M., Poursaleh A., Ghasempour G., Farhad S., Najafi M. (2019). The effects of oxidative stress on the development of atherosclerosis. Biol Chem.

[16] Brown J.C., Gerhardt T.E., Kwon E. (2023). StatPearls. Treasure island (FL).

[17] Rafieian-Kopaei M., Setorki Baradaran A., Nasri H. (2014). Atherosclerosis: process, indicators, risk factors and new hopes. Int J Prev Med.

[18] Bentzon J.F., Otsuka F., Virmani R., Falk E. (2014). Mechanisms of plaque formation and rupture. Circ Res.

[19] Frantz S., Bauersachs J., Ertl G. (2009). Post-infarct remodeling: contribution of wound healing and inflammation. Cardiovasc Res.

[20] Jaiswal Y.S., Williams L.L. (2016). A glimpse of Ayurveda - the forgotten history and principles of Indian traditional medicine. J Tradit Complement Med.

[21] Prasher B., Negi S., Aggarwal S., Mandal A.K., Sethi T.P., Deshmukh S.R. (2008). Whole genome expression and biochemical correlates of extreme constitutional types defined in Ayurveda. J Transl Med.

[22] Prasher B., Gibson G., Mukerji M. (2016). Genomic insights into ayurvedic and western approaches to personalized medicine. J Genet.

[23] Tiwari P., Kutum R., Sethi T., Girase B., Aggarwal S., Patil R. (2017). Recapitulation of Ayurveda constitution types by machine learning of phenotypic traits. PLoS One.

[24] Mahalle N.P., Kulkarni M.V., Pendse N.M., Naik S.S. (2012). Association of constitutional type of Ayurveda with cardiovascular risk factors, inflammatory markers and insulin resistance. J Ayurveda Integr Med.

[25] Thomas M., Jones P.G., Arnold S.V., Spertus J.A. (2021). Interpretation of the Seattle angina questionnaire as an outcome measure in clinical trials and clinical care: a review. JAMA Cardiol.

[26] Tabet J.Y., Malergue M.C., Guenoun M., Paganelli F., Meurin P., Not D. (2010). Distribution of left ventricular ejection fraction and heart rate values in a cohort of stable coronary patients: the INDYCE registry. Arch Cardiovasc Dis.

[27] Minczykowski A., Zwanzig M., Dziarmaga M., Rutkowska A., Baliński M., Krauze T. (2023). First- phase left ventricular ejection fraction as an early sign of left ventricular dysfunction in patients with stable coronary artery disease. J Clin Med.

[28] Lawal O.A., Awosoga O., Santana M.J., James M.T., Wilton S.B., Norris C.M. (2022). Measurement invariance of the Seattle Angina Questionnaire in coronary artery disease. Qual Life Res.

[29] Roth Gregory A., Abate Degu, Hassen Abate Kalkidan, Abay Solomon M., Abbafati Cristiana, Abbasi Nooshin (2019). Causes of Death Collaborators. Global, regional, and national age-sex-specific mortality for 282 causes of death in 195 countries and territories, 1980-2017: a systematic analysis for the Global Burden of Disease Study. Lancet.

[30] (2021). ICMR guidelines for good clinical laboratory practices (GcLP).

[31] Ji H., Li Y., Fan Z., Zuo B., Jian X., Li L. (2017). Monocyte/lymphocyte ratio predicts the severity of coronary artery disease: a syntax score assessment. BMC Cardiovasc Disord.

[32] Homma Y. (2004). Predictors of atherosclerosis. J Atherosclerosis Thromb.

[33] Guo Q., Feng X., Zhang B., Zhai G., Yang J., Liu Y. (2022). Influence of the triglyceride-glucose index on adverse cardiovascular and cerebrovascular events in prediabetic patients with acute coronary syndrome. Front Endocrinol.

[34] Somani B.L., Arora M.M., Datta S.K., Negi R., Gupta A. (2013). Prevalence of unsuspected glucose intolerance in coronary artery disease (CAD) patients: importance of HbA1c. Med J Armed Forces India.

[35] Doganer Y.C., Rohrer J.E., Aydogan Agerter D.C., Cayci T., Barcin C. (2015). Atherosclerosis and liver function tests in coronary angiography patients. West Indian Med J.

[36] Shroff G.R., Carlson M.D., Mathew R.O. (2021). Coronary artery disease in chronic kidney disease: need for a heart-kidney team-based approach. Eur Cardiol.

[37] Shrivastava A.K., Singh H.V., Raizada A., Singh S.K. (2015). C-reactive protein, inflammation, and coronary heart disease. Egypt Heart J.

[38] Wainstein M.V., Mossmann M., Araujo G.N., Gonçalves S.C., Gravina G.L., Sangalli M. (2017). Elevated serum interleukin-6 is predictive of coronary artery disease in intermediate risk overweight patients referred for coronary angiography. Diabetol Metab Syndr.

[39] Gao J., Yang Y.N., Cui Z., Feng S.Y., Ma J., Li C.P. (2021). PCSK9 is associated with severity of coronary artery lesions in male patients with premature myocardial infarction. Lipids Health Dis.

[40] Sairam S.G., Sola S., Barooah A., Javvaji S.K., Jaipuria J., Venkateshan V. (2017). The role of Lp-PLA2 and biochemistry parameters as potential biomarkers of coronary artery disease in Asian South-Indians: a case-control study. Cardiovasc Diagn Ther.

[41] Boaz M., Matas Z., Biro A., Katzir Z., Green M., Fainaru M. (1999). Serum malondialdehyde and prevalent cardiovascular disease in hemodialysis. Kidney Int.

[42] Firoozrai M., Mehrabi H., Ehsani A., Najafi M., Ghaffari M. (2007). Activities of anti-oxidative enzymes, catalase, and glutathione reductase in red blood cells of patients with coronary artery disease. Asian J Biochem.

[43] Abdel-Aal Fadel Monazzama, Abdel-Hameed Kafafy Tarek, Essam Salma M., Amal M Abdel-Aal (2022). Serum superoxide dismutase and malondialdehyde as oxidative stress biomarkers in coronary artery disease. J Curr Medical Res Pract.

[44] Sakai H., Tsutamoto T., Ishikawa C., Tanaka T., Fujii M., Yamamoto T. (2007). Direct comparison of brain natriuretic peptide (BNP) and N-terminal pro-BNP secretion and extent of coronary artery stenosis in patients with stable coronary artery disease. Circ J.

[45] Hajar R. (2017). Risk factors for coronary artery disease: historical perspectives. Heart Views.

[46] Senoner T., Dichtl W. (2019). Oxidative stress in cardiovascular diseases: still a therapeutic target?. Nutrients.

[47] Weidner G. (2000). Why do men get more heart disease than women? An international perspective. J Am Coll Health.

[48] Gerke O., Lindholt J.S., Abdo B.H., Lambrechtsen J., Frost L., Steffensen F.H. (2022). Prevalence and extent of coronary artery calcification in the middle-aged and elderly population. Eur J Prev Cardiol.

[49] Pednekar M.S., Gupta R., Gupta P.C. (2011). Illiteracy, low educational status, and cardiovascular mortality in India. BMC Public Health.

[50] Salehi N., Janjani P., Tadbiri H., Rozbahani M., Jalilian M. (2021). Effect of cigarette smoking on coronary arteries and pattern and severity of coronary artery disease: a review. J Int Med Res.

[51] Ayhan S., Ozturk S., Erdem A., Ozlu M.F., Memioglu T., Ozyasar M. (2013). Hematological parameters and coronary collateral circulation in patients with stable coronary artery disease. Exp Clin Cardiol.

[52] Madjid M., Fatemi O. (2013). Components of the complete blood count as risk predictors for coronary heart disease: in-depth review and update. Tex Heart Inst J.

[53] Sweetnam P.M., Thomas H.F., Yarnell J.W., Baker I.A., Elwood P.C. (1997). Total and differential leukocyte counts as predictors of ischemic heart disease: the Caerphilly and Speedwell studies. Am J Epidemiol.

[54] Ensrud K., Grimm R.H. (1992). The white blood cell counts and risk for coronary heart disease. Am Heart J.

[55] Mehta J.L., Nichols W.W., Mehta P. (1988). Neutrophils as potential participants in acute myocardial ischemia: relevance to reperfusion. J Am Coll Cardiol.

[56] Khandekar M.M., Khurana A.S., Deshmukh S.D., Kakrani A.L., Katdare A.D., Inamdar A.K. (2006). Platelet volume indices in patients with coronary artery disease and acute myocardial infarction: an Indian scenario. J Clin Pathol.

[57] Naito R., Kasai T. (2015). Coronary artery disease in type 2 diabetes mellitus: recent treatment strategies and future perspectives. World J Cardiol.

[58] Majid A. (2009). Prevention and management of coronary artery disease in patients with diabetes mellitus. Acta Med Indones.

[59] Wassmann S., Stumpf M., Strehlow K., Schmid A., Schieffer B., Böhm M. (2004). Interleukin- 6 induces oxidative stress and endothelial dysfunction by overexpression of the angiotensin II type 1 receptor. Circ Res.

[60] Gaweł S., Wardas M., Niedworok E., Wardas P. (2004). [Malondialdehyde (MDA) as a lipid peroxidation marker. Wiad Lek.

[61] Nandi A., Yan L.J., Jana C.K., Das N. (2019). Role of catalase in oxidative stress- and age- associated degenerative diseases. Oxid Med Cell Longev.

[62] Aggarwal S., Gheware A., Agrawal A., Ghosh S., Prasher B., Mukerji M. (2015). Combined genetic effects of EGLN1 and VWF modulate thrombotic outcome in hypoxia revealed by Ayurgenomics approach. J Transl Med.

[63] Abbas T., Chaturvedi G., Prakrithi P., Pathak A.K., Kutum R., Dakle P. (2022). Whole exome sequencing in healthy individuals of extreme constitution types reveals differential disease risk: a novel approach towards predictive medicine. J Personalized Med.

[64] Cammisotto V., Baratta F., Simeone P.G., Barale C., Lupia E., Galardo G. (2022). Proprotein convertase subtilisin kexin type 9 (PCSK9) beyond lipids: the role in oxidative stress and thrombosis. Antioxidants (Basel).

[65] Rani R., Rengarajan P., Sethi T., Khuntia B.K., Kumar A., Punera D.S. (2022). Heart rate variability during head-up tilt shows inter-individual differences among healthy individuals of extreme Prakriti types. Phys Rep.

[66] Dua P., Seth S., Prasher B., Mukerji M., Maulik S.K., Reeta K.H. (2024). Pharmacogenomic biomarkers in coronary artery disease: a narrative review. Biomarkers Med.

[67] Khatua D., Sekh A.A., Kutum R., Mukherji M., Prasher B., Kar S. (2023). Classification of Ayurveda constitution types: a deep learning approach. Soft Comput.

